# Parasite communities in English Sole (*Parophrys vetulus*) have changed in composition but not richness in the Salish Sea, Washington, USA since 1930

**DOI:** 10.1017/S0031182022000233

**Published:** 2022-05

**Authors:** Whitney C. Preisser, Rachel L. Welicky, Katie L. Leslie, Natalie C. Mastick, Evan A. Fiorenza, Katherine P. Maslenikov, Luke Tornabene, John M. Kinsella, Chelsea L. Wood

**Affiliations:** 1 School of Aquatic and Fishery Sciences, University of Washington, Seattle, WA, USA; 2 Unit for Environmental Sciences and Management, North–West University, Potchefstroom, South Africa; 3 Ecology and Evolutionary Biology, University of California, Irvine, Irvine, CA, USA; 4 Burke Museum Ichthyology Collection, University of Washington, Seattle, WA, USA; 5 HelmWest Laboratory, Missoula, MT, USA

**Keywords:** Alpha diversity, beta diversity, biodiversity, ecology

## Abstract

Earth is rapidly losing free-living species. Is the same true for parasitic species? To reveal temporal trends in biodiversity, historical data are needed, but often such data do not exist for parasites. Here, parasite communities of the past were reconstructed by identifying parasites in fluid-preserved specimens held in natural history collections. Approximately 2500 macroparasites were counted from 109 English Sole (*Parophrys vetulus*) collected between 1930 and 2019 in the Salish Sea, Washington, USA. Alpha and beta diversity were measured to determine if and how diversity changed over time. Species richness of parasite infracommunities and community dispersion did not vary over time, but community composition of decadal component communities varied significantly over the study period. Community dissimilarity also varied: prior to the mid-20th century, parasites shifted in abundance in a seemingly stochastic manner and, after this time period, a canalization of community change was observed, where species' abundances began to shift in consistent directions. Further work is needed to elucidate potential drivers of these changes and to determine if these patterns are present in the parasite communities of other fishes of the Salish Sea.

## Graphical abstract

**Figure:**
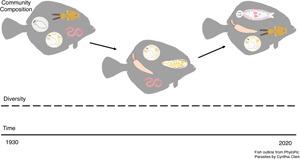

## Introduction

Ecosystems are losing biodiversity at rates that match mass extinctions (Barnosky *et al*., 2011). This is an especially urgent problem for parasitic taxa, which may account for up to 40% of the animal biodiversity in an ecosystem (Dobson *et al*., 2008). Conservative models estimate that up to 10% of parasite species will be driven extinct by 2070 due to habitat loss from climate change alone (Carlson *et al*., 2017), and this increases to 30% when accounting for secondary extinctions (when parasites diminish to extinction alongside their hosts; Colwell *et al*., 2012). Although it might seem counterintuitive to consider parasites as a conservation target, parasitic species are essential to ecosystem function (Wood and Johnson, 2015). They are involved in up to 78% of trophic links in some food webs (Lafferty *et al*., 2006), and can structure communities, influence host population dynamics, drive host diversification and shape energy flow in an ecosystem (Hudson *et al*., 2006; Wood and Johnson, 2015).

As ecosystems face increasing anthropogenic modification, singular ‘snapshots’ of biodiversity reveal little about how diversity changes through time and in response to human impacts (Mushet *et al*., 2019). Long-term datasets allow ecologists to establish baseline diversity and, potentially, to separate natural changes from those driven by anthropogenic causes. However, data availability remains one of the biggest barriers to studying temporal trends of biodiversity (Magurran *et al*., 2019; Mushet *et al*., 2019). Temporal datasets exist for many free-living taxa. For example, Dornelas *et al*. (2018) compiled an open access dataset comprising over 8.7 million abundance records for 44 440 species between 1874 and 2016 (BioTIME). With these data, long-term trends in diversity, species composition and abundance can be analysed in marine, freshwater and terrestrial environments.

Unfortunately, long-term datasets for parasitic species are scarce, as these taxa are rarely documented in historical datasets (Harmon *et al*., 2019) and little is known about parasite diversity in general (Carlson *et al*., 2020). Aguirre-Macedo *et al*. (2011) sampled parasite diversity in snails from 2001 to 2009 to study the effects of hurricanes on trematode communities. May-Tec *et al*. (2013) and Vidal-Martínez *et al*. (2014) sampled parasites from a cichlid over an 8- and 5-year period, respectively, to understand the influence of rainfall and temperature on parasite prevalence, abundance and aggregation. While Howard *et al*. (2019) and Welicky *et al*. (2021) measured parasite abundance in English Sole (*Parophrys vetulus*) over 90 years, to our knowledge, there are no temporally comparable studies investigating historical trends in parasite diversity.

Recently, novel approaches have unlocked new techniques for generating data on historical parasite infections (Harmon *et al*., 2019; Howard *et al*., 2019; Fiorenza *et al*., 2020). By examining fluid-preserved specimens, disease ecologists can reconstruct nearly complete parasite assemblages because both larval and adult parasites are preserved alongside their host (Fiorenza *et al*., 2020). Recent experiments have validated the approach of using parasitological dissection to assess the number and identity of parasites infecting a fluid-preserved animal at the time of its death (Fiorenza *et al*., 2020) and this methodology has already been successfully used to assess temporal change in parasite abundance (Murphy *et al*., 2020; Welicky *et al*., 2021). However, this approach has never before been used to assess temporal change in parasite biodiversity and community structure.

Fluid-preserved English Sole (*P. vetulus*) specimens held in the University of Washington's Burke Museum Fish Collection (UWFC) were sampled to assemble a historical dataset of macroparasite communities in the Salish Sea, Washington, USA and to describe long-term trends in parasite diversity. Over the past century, English Sole have been an economically important and abundant species in the Salish Sea. They are well-represented in natural history collections, with over 4000 English Sole collected since the 1920s held in UWFC alone (Fishnet2, 2017). With these resources, the longest historical dataset on the diversity of parasites for any wildlife host was assembled.

**Fig. A1.**
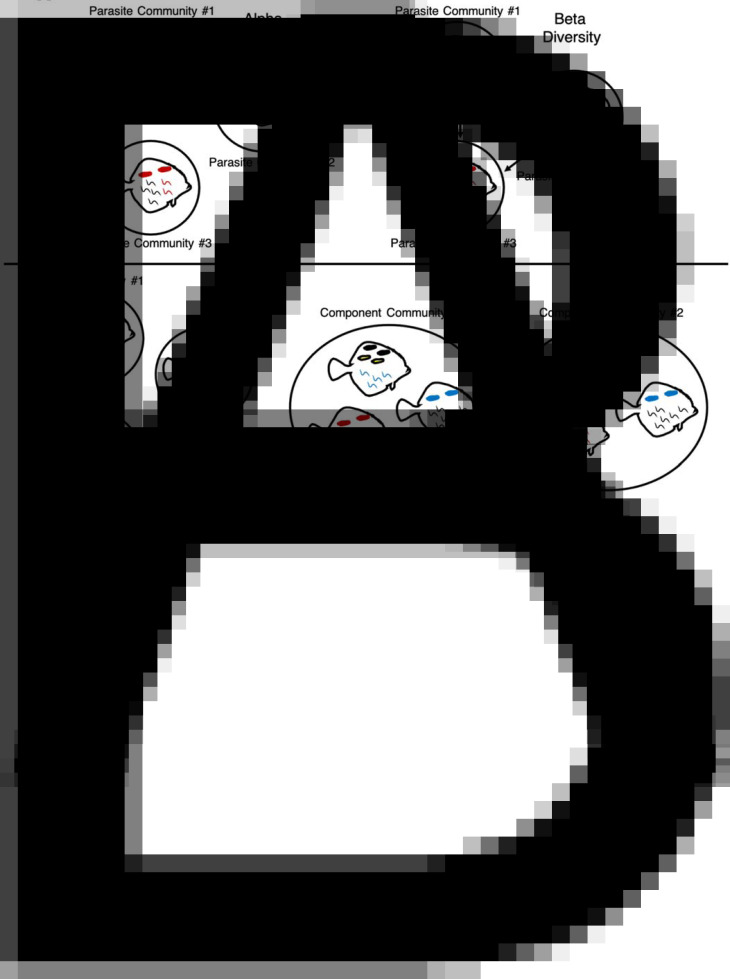
Visual representations of parasite alpha and beta diversity (A) and infracommunity and component community definitions (B). Alpha diversity describes the diversity of a local community, while beta diversity describes the changes in species and abundance between communities (Whittaker, 1960). Parasite communities can be defined at multiple levels; in this study, we defined parasite communities at two scales following Bush *et al*. (1997), at the level of infracommunity (all of the parasites on or in an individual fish host) and component community (all of the parasites associated with a subset of a host species or, in this case, all of the parasites associated with hosts collected within a particular decade).

Large changes in parasite diversity over time were expected, given the anthropogenic pressures that have affected the Salish Sea over the past century, like pollution from inorganic and organic compounds (Brandenberger *et al*., 2008), fishing pressure (Stewart, 2005) and climate change (Department of Fish and Oceans Canada, 2017). However, before one can begin to understand how these disturbances might impact parasite communities, historical trends in diversity first need to be described. To do this, trends in alpha and beta diversity of parasite communities between 1930 and 2019 were investigated. Alpha diversity reflects the diversity of a local assemblage or community, and beta diversity describes the differences between assemblages across time or space (Whittaker, 1960; Appendix Fig. A1). Temporal trends in diversity were investigated at the level of the individual host, where each host has an infracommunity of parasites, and at the level of decade, where parasite communities of host individuals from each decade were grouped into decadal component communities (Bush *et al*., 1997; Fig. A1). Analysing diversity at these two scales allowed for the exploration of how diversity changes at both the individual and host population levels. We therefore sought to answer the following questions for parasite communities in English Sole:

1. How has the alpha diversity of parasite infracommunities changed over the past nine decades?
2. To address how the beta diversity of host communities has changed:
  - Do decades vary in their parasite assemblages?
  - How has the composition of parasite assemblages changed across time?

## Methods

### Data collection

**Fig. A2.**
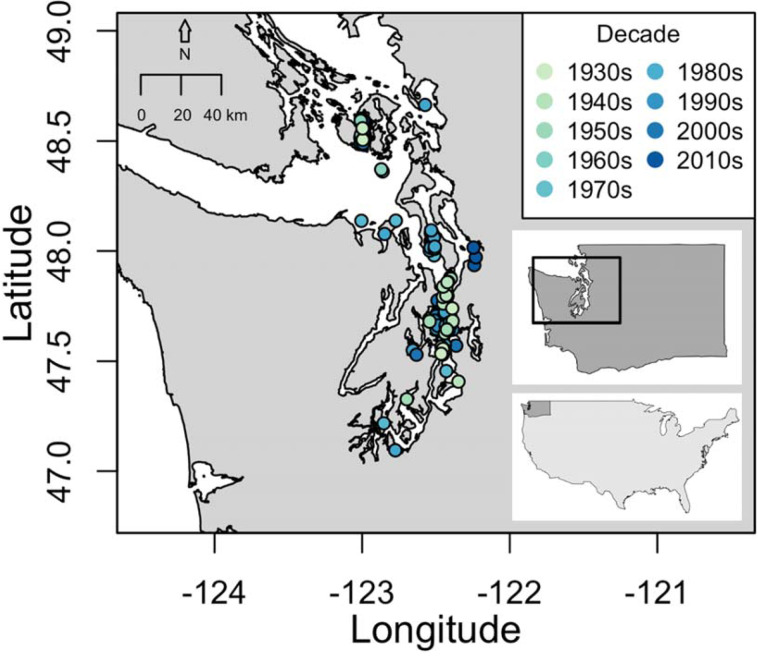
A map of the collection localities in Puget Sound and the larger Salish Sea where English Sole were collected between 1930 and 2019. Localities are colour coded by the decade in which they were collected.

One hundred and nine English Sole (*P. vetulus*) collected in the Salish Sea between 1930 and 2019 and held at the UWFC were dissected to identify their parasites (Appendix Fig. A2). Because sampling is partially destructive, a maximum of 16 individuals per decade was sampled to avoid compromising availability of intact specimens for future museum loans. Individuals that were close to the average size of all of the English Sole in the UWFC (SL = 164.480 ± 53.294 mm) were chosen; if more than 16 suitably sized individuals were available per decade, individuals were haphazardly chosen from among those of suitable size. Each fish was dissected and their ecto- and endoparasites collected. Parasites were sorted by morphotype, counted, identified using standard keys and preserved according to standard methods (Appendix A2).

All analyses were performed in R v4.0.0 (R Core Team, 2020). The dataset and R code are available at https://github.com/wood-lab/Preisser-et-al.-Parasitology2022.

#### How has the alpha diversity of host communities changed over the past nine decades?

Changes in the alpha diversity of parasite infracommunities over time were detected by analysing the relationship between time and species richness. Species richness was chosen because it is a common measure of alpha diversity. The parasite species richness value was compiled for each parasite infracommunity and used as the response variable in all models. As parasite diversity might be more similar in fish collected in close spatial proximity or time, spatial and temporal autocorrelations that could violate the assumptions of the statistical models were tested for in the dataset (Appendix A2). How the alpha diversity of infracommunities varied over time was analysed using a generalized linear mixed-effects model with penalized quasi-likelihood (‘glmmPQL’ function in ‘MASS’ package; Venables and Ripley, 2002), to allow for the inclusion of a correlation structure term if spatial or temporal autocorrelation was present, and a Poisson distribution, as is appropriate for count data that are not overdispersed (Appendix A2). Host body size was also included as a covariate, given that host body size is often correlated with parasite richness and abundance (Poulin, 1997), and collection locality was included as a random intercept (equation (1)). As changes in parasite species richness may not be linear across time, these data were also analysed with a fourth order polynomial regression; the results were the same between the mixed-effects model and the polynomial regression, so only the mixed-effects model is reported here. To ensure that any non-significant result for the effect of time was not due to low statistical power, a power analysis using a simulation approach was conducted (Appendix A2).1
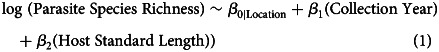


#### Do decades vary in their parasite assemblages?

First, rarefaction and extrapolation curves were assembled to determine if the sampling sufficiently captured the parasite diversity within each decade (Appendix A2). Decades were considered sufficiently sampled if the observed richness was two or fewer species less than the estimated richness, and only sufficiently sampled decades were included in the beta diversity analyses.

As communities can change in composition and abundance without changing the number of species present (Hillebrand *et al*., 2018), parasite communities within each decade were analysed to determine if they significantly varied in their community composition or dispersion (i.e. the variance among infracommunities) over time. Using a Bray–Curtis dissimilarity matrix (Appendix A2), a PERMANOVA was run to test whether the community compositions of decades were significantly different (‘adonis’ function in ‘vegan’ package; Oksanen *et al*., 2019). To determine whether the dispersion of the infracommunities within each decade was significantly different across decades, a multivariate homogeneity of group dispersions (‘betadisper’ function in ‘vegan’ package; Oksanen *et al*., 2019) and an analysis of variance (‘anova’ function in ‘stats’ package; R Core Team, 2020) were run. The infracommunities of the decades were plotted using a principal coordinate analysis and the ‘betadisper’ output. The centroids of each decade (including the 1960s and 1970s as there was no significance testing for this plot) were also plotted in multivariate space to observe the change in communities over time.

#### How has the composition of parasite assemblages changed across time?

**Fig. A3.**
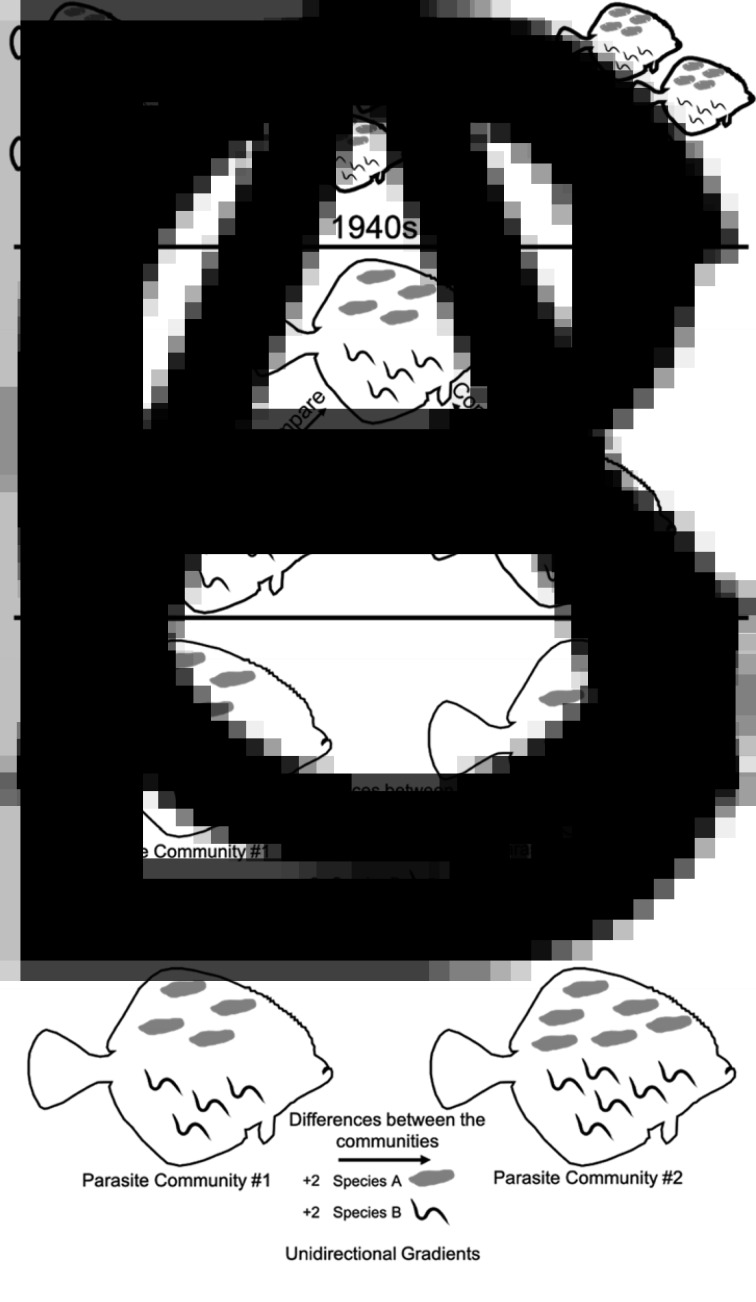
To determine how parasite assemblages have changed over the past 90 years, we assessed how the parasite community dissimilarity between decadal parasite component communities (A, where we grouped parasite communities from individual fish within each decade) and among infracommunities within each decade (B) varied over time. Using pairwise dissimilarities between successive decades (A) and multiple site dissimilarities among individual communities (B), we calculated the total dissimilarity as well as its two components, unidirectional gradients and balanced variation (C). Unidirectional gradients describe the equal increase or decrease in abundance across all species between sites or communities. Balanced variation describes the decline in abundance of some species and the equal increase in abundance of other species between sites. These components are not mutually exclusive and can occur concurrently. Figure modified from Baselga (2013*a*).

While the analyses described above sought to determine *if* communities significantly varied in their composition and dispersion, these next methods determined *how* communities varied over time. Both the decadal component community composition and the infracommunity composition within each decade were examined over the past nine decades to determine how community composition changed over time (Appendix Fig. A3). The community composition between decades (e.g. 1930s–1940s, 1940s–1950s, etc.) was compared using pairwise dissimilarities (‘beta.pair.abund’ function in ‘betapart’ package; Baselga *et al*., 2018; matrices described in Appendix A2). For each decade pair, total dissimilarity (Bray–Curtis) as well as its two abundance-based components, balanced variation and unidirectional gradients (Appendix Fig. A3), were calculated.

Balanced variation describes the equal changes in abundance between communities, with some species increasing in abundance and others decreasing in equal measure; unidirectional gradients describe equal increases or decreases in abundance of all species between communities (Baselga, 2013*a*). For the total dissimilarity, a dissimilarity score of 0 means that communities are identical while a dissimilarity score of 1 means communities are completely dissimilar. The dissimilarity scores of the two components (balanced variation and unidirectional gradients) add together to equal the total dissimilarity score. For the two components, a score of 0 means that that component is not contributing to observed differences between communities, while a score equal to the total dissimilarity measure means that that component is solely driving the observed differences. At intermediate values of balanced variation and unidirectional changes in abundance, both mechanisms are contributing to observed dissimilarity between communities (Baselga, 2013*a*).

How the dissimilarity of parasite communities of individual hosts within each decade varied between sequential decades was assessed to determine how parasite infracommunities within each decade became more or less dissimilar over time. The dissimilarities (total *via* Bray–Curtis, balanced variation and unidirectional gradients) within each decade were calculated using multiple site dissimilarity (‘beta.multi.abund’ function in ‘betapart’ package; Baselga *et al*., 2018; matrices described in Appendix A2). Because it accounts for patterns of co-occurrence across all sites rather than between pairs of sites, multiple site dissimilarity can better quantify overall heterogeneity among communities (Baselga, 2013*b*).

## Results

### Data collection

Specimen availability limited the number of hosts available from each decade (1930s: *n* = 14; 1940s: *n* = 14; 1950s: *n* = 6; 1960s: *n* = 8; 1970s: *n* = 11; 1980s: *n* = 13; 1990s: *n* = 13; 2000s: *n* = 16; 2010s: *n* = 14). Across the 109 individuals dissected, at least 23 parasite taxa and 2450 individual parasites were collected (excluding the single taxon recorded only as presence/absence; Data S4), including eight species of nematodes, one cestode, eight trematodes, at least one species of acanthocephalan, four copepods and one leech (Appendix Table A1); acanthocephalans could not be identified to species and may represent one or more species. Individual hosts had an average of 2.65 parasite species (range: 0–6 species). Three hundred and fifty-three individual parasites could not be identified and were excluded from analyses (Appendix Table A1), with the exception of the three immature trematodes included in the alpha diversity analysis. Four of the 109 fish sampled had no parasites.

#### How has alpha diversity of host communities changed over the past nine decades?

**Fig. A4.**
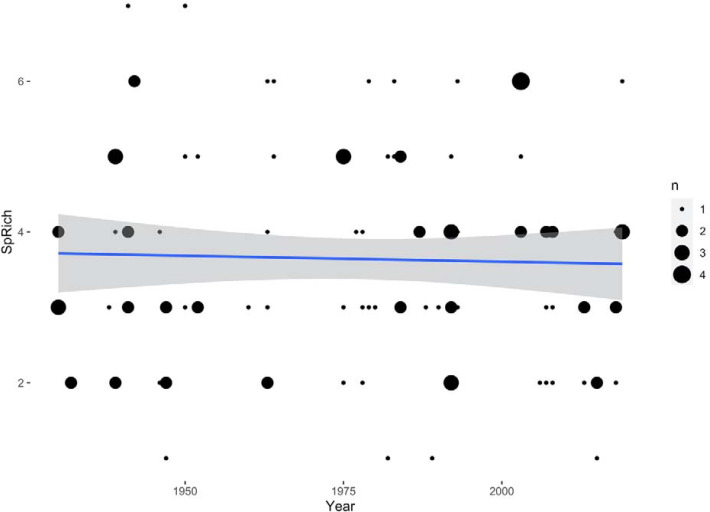
A plot showing the lack of relationship between parasite species richness (SpRich) and collection year (Year) with a predicted regression line and a 95% confidence interval. The size of the points corresponds to the number of infracommunities at that time period with that richness value.

**Fig. A5.**
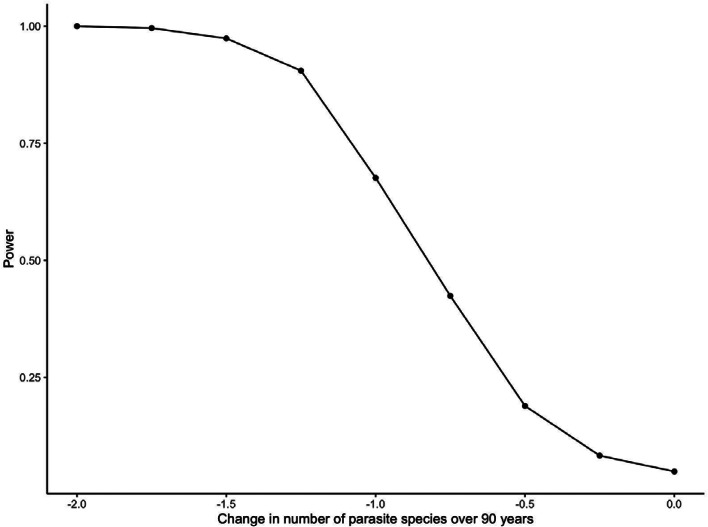
Observed power from a simulation-based power analysis for differing levels of change in average parasite species richness over 90 years. Here, power is defined as the proportion of simulations in which a model for containing ‘year’ outperformed a null model based on Akaike information criterion for a sample size of 109. We kept the same sample size and observations per year as the real data, and only varied the parasite richness of each host. At 80% power, it is possible to detect the loss of ~1.25 parasite species. Points are simulated scenarios and the line connects the points to aid in visualization.

Species richness was not significantly correlated with time (estimate ± standard error = −0.00161 ± 0.00216, *t* value = −0.748, *P* value = 0.457; Appendix Fig. A4 and Table A2). Species richness increased with host body size (estimate ± standard error = 0.00320 ± 0.00151, *t* value = 2.12, *P* value = 0.0371). Power analysis determined that the model had sufficient (i.e. >80%) statistical power to detect a loss in parasite richness that exceeded 1.25 species over the 90-year period (Appendix Fig. A5).

#### Do decades vary in their parasite assemblages?

**Fig. A6.**
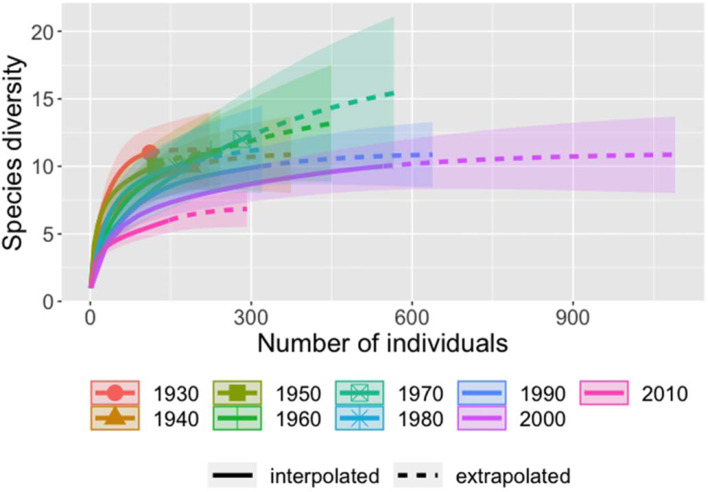
Rarefaction curves for the component parasite community within each decade. The *x*-axis represents the number of parasite individuals. Parasite infracommunities from individual fish within each decade were combined into one component community per decade and a rarefaction curve was created for each decade (Table A3; ‘iNEXT’ function in the ‘iNEXT’ package; Chao *et al*., 2014, Hsieh *et al*., 2020).

Using rarefaction and extrapolation curves (Appendix Fig. A6 and Table A3), it was found that all but two decades had observed diversity within two species of the estimated diversity. The curves for the 1960s and 1970s did not near the horizontal asymptote, suggesting that replication was too low to obtain good representation of the parasite communities in those decades. These two decades were excluded from the beta diversity analyses for this reason.

**Fig. 1.**
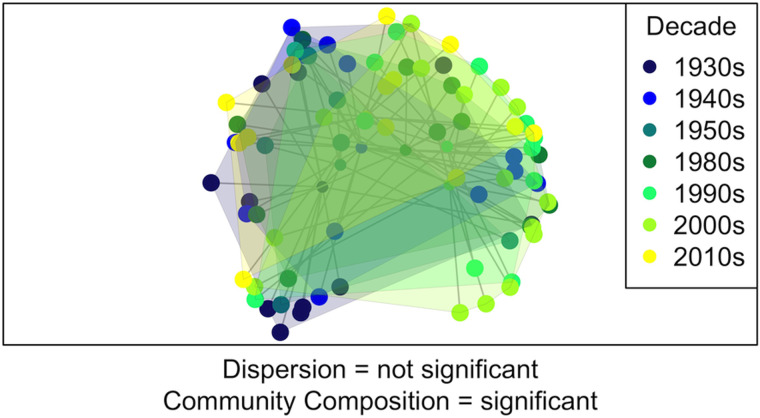
Parasite communities from seven decades in multivariate space using a principal coordinates analysis. Each point represents a parasite community from an individual fish and is colour-coded according to the decade in which the fish was collected. While the decades did not differ significantly in the dispersion of their parasite communities, they did significantly differ in their parasite compositions. Axis 1 (*x*-axis) explains 27.46% of the variation while axis 2 (*y*-axis) explains 18.6% of the variation.

**Fig. A7.**
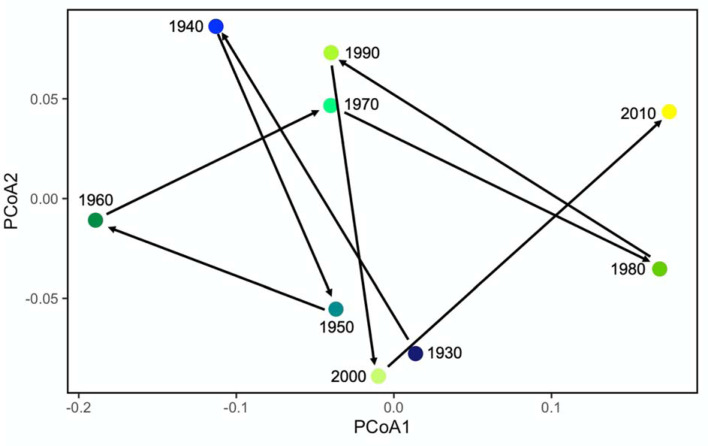
The centroids of the parasite component communities of each decade in multivariate space using a principal coordinates analysis and connected along a timeline. Unlike Fig. 2, this figure includes parasite communities from the 1960s and 1970s. Figure modelled after Aguirre-Macedo *et al*. (2011).

**Fig. A8.**
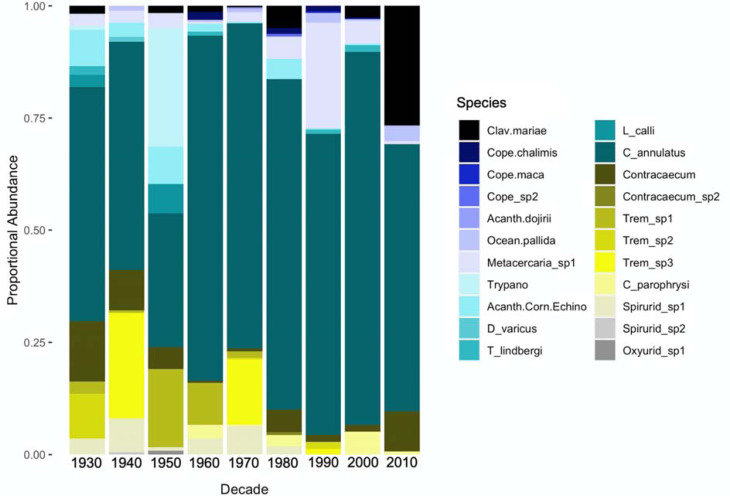
The component community composition and proportional abundance of each species within each decade. Twenty-two of the 23 taxa are shown; Metacercaria sp. 2 was excluded from this figure as it lacked abundance data and was recorded as presence/absence only. Please refer to Welicky *et al*. (2021) to see the analyses of changes in abundance for each taxon and higher taxonomic group. Whole species names are given in Table A1.

When the dispersion and community composition of infracommunities within each decade (except the 1960s and 1970s) were compared, decades differed significantly in composition (PERMANOVA, *F* statistic = 3.834, *R*^2^ = 0.0447, *P* value = 0.002; Table A4, Fig. 1) but not dispersion (*F* statistic = 1.851, *P* value = 0.100). Decadal component communities plotted in multivariate space did not follow a particular pattern (Appendix Fig. A7); instead, component communities varied greatly from decade to decade. The specific community composition of each decadal component community is represented in Appendix Fig. A8.

#### How has the composition of parasite assemblages changed across time?

**Fig. 2.**
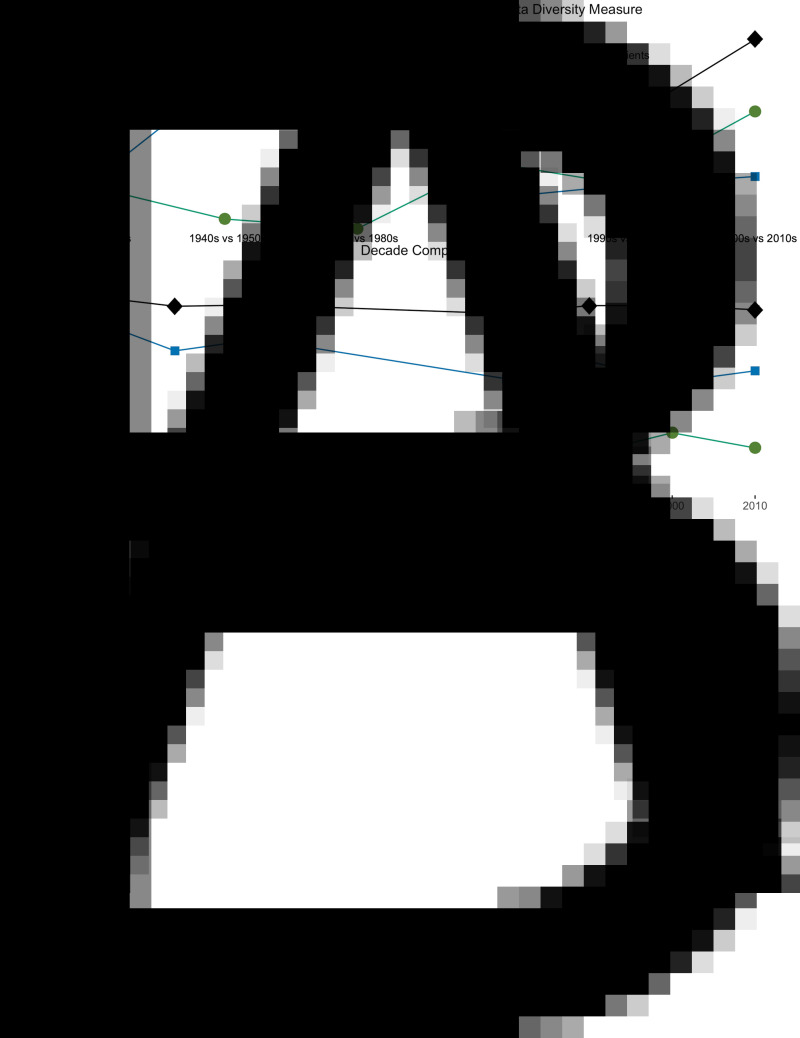
Total dissimilarity and its two components, balanced variation and unidirectional gradients, of (A) parasite communities between successive decades (with the exception of 1950s and 1980s) and (B) among parasite communities within a decade. For (A), parasite communities from individuals within each decade were pooled, and successive decades were compared using pairwise dissimilarity with parasite abundance. For (B), total dissimilarity and its two components were calculated using multiple site dissimilarity with parasite abundances among all individuals within a decade.

Comparing the aggregated parasite communities between successive decades, total dissimilarity between decades ranged from 0.412 to 0.676 (Appendix Table A5, Fig. 2A). Between the earlier decades, community dissimilarity was primarily driven by balanced variation (i.e. some species increased between communities and others decreased in equal measure); in later decades, community dissimilarity was primarily driven by unidirectional gradients (i.e. all species increased or decreased between communities; Appendix Table A5 and Fig. A3, Fig. 2A).

When the infracommunities within each decade were compared, community dissimilarity was high (total dissimilarity ranged from 0.855 to 0.931; Appendix Table A6, Fig. 2B), suggesting very dissimilar infracommunities within each decade. Of the two beta diversity components, balanced variation was consistently higher than unidirectional gradient changes across all decades (Appendix Table A6, Fig. 2B), such that changes between parasite communities were primarily driven by equivalent increases and decreases in abundance.

## Discussion

The alpha diversity of individual English Sole parasite communities did not change between 1930 and 2019 in the Salish Sea. We are confident that the lack of change in parasite richness over time was not due to an underpowered sampling design, given that power analysis indicated sufficient power to detect a loss of more than 1.25 species over the study period. Diversity measures that compare species identity and abundance between assemblages are more sensitive to changes over time than are measures of species richness (Hillebrand *et al*., 2018). Indeed, previous studies have demonstrated a lack of change in species richness accompanying detectable changes in community assemblages (Hillebrand *et al*., 2018). Here, parasite community composition significantly differed across the 1930s, 1940s, 1950s, 1980s, 1990s, 2000s and 2010s, though the dispersion of community assemblages did not significantly differ. Similarly, differences in how parasite communities were structured were observed: prior to the mid-1900s, differences between communities were characterized by some species increasing in abundance while others were decreasing; after the mid-point of the 20th century, species abundances changed in a more uniform way.

Previous work in this same host–parasite system noted that only three taxa significantly changed in abundance over the sampling period (Welicky *et al*., 2021). The relatively low amount of change in both diversity and abundance over time is surprising given the Salish Sea's history of anthropogenic disturbances (e.g. Stewart, 2005; Brandenberger *et al*., 2008; Department of Fish and Oceans Canada, 2017) and given that such disturbances can alter parasite communities (Wood and Johnson, 2015). Heavy metals were a major source of pollution in Puget Sound, the southern part of the Salish Sea, beginning in the 1890s (Brandenberger *et al*., 2008). However, with the passage of the Clean Water Act in 1972, pollution levels have decreased in the area, with some metal concentrations expected to decline to preindustrial concentrations by 2035 (Brandenberger *et al*., 2008). English Sole are often used as sentinels in contamination studies and contaminants and pollutants are detected in their tissues today (Moser *et al*., 2013). Additionally, from 1917 to the 1950s, English Sole were considered high value targets in Puget Sound and were fished almost exclusively with bottom trawl gear, with peaks in hauls occurring during the 1920s and 1940s to the 1960s (Stewart, 2005). Beginning in the 1980s, sole were indirectly protected from overfishing due to trawling bans (Palsson *et al*., 1996, 2009).

Anthropogenic disturbances can impact parasites in complex ways (Lafferty and Kuris, 2007); for example, the same disturbance can have different impacts on parasites with direct life cycles (i.e. only one host species is required for development and reproduction) *vs* those with complex life cycles (i.e. more than one host species is required). Wood and Lafferty (2015) found that, in response to fishing pressure, the abundance of complex life-cycle parasites in fished hosts decreased, while there was no response in directly transmitted parasites. As complex life-cycle parasites use multiple host species, and their final host is often at a high trophic level, the chance of their life cycle being disrupted by fishing (or another anthropogenic disturbance) may be greater than for directly transmitted parasites.

The composition of individual host communities within each decade was significantly different across the decades; that is, different taxa were detected through time (Appendix Fig. A8). In some cases, parasite species may have been present in English Sole during these decades and were simply not present in the fish sampled, or some species may have been extirpated from particular collection localities; in other cases, parasites may have spread to new localities during the sampling period and these changes in distributions were picked up by the analyses. The parasite taxa collected have both complex and direct life cycles and demonstrate no clear patterns of presence or absence corresponding to life-cycle type, host species use or higher taxonomic group. Similarly, with an exception for the Acanthocephala, Welicky *et al*. (2021) found no correlation between life cycle or taxonomic group and changes in abundance of English Sole parasites. Future work should identify natural and anthropogenic disturbances and investigate the routes through which they alter parasite communities of English Sole in the Salish Sea.

Even in the absence of significant changes in community composition and dispersion, distinct changes in dissimilarity were seen across multiple analyses between the 1950s and the 1980s, suggesting a substantial environmental or host-related change that impacted parasite community structure in the Salish Sea. Prior to this time period, parasite species were increasing and decreasing; there were no major directional changes in species abundances between communities. However, after this point, differences among individual communities and between decadal communities were increasingly due to consistent, directional changes across species (Fig. 1).

The increasing homogenization of community change suggests a major disturbance. Prior to this event, communities were changing in a seemingly stochastic manner, and afterwards, changes in species' abundances became more positively correlated with each other: there was a canalization of community change where species' abundances began to shift in a similar manner. Both anthropogenic and natural disturbances have been found to homogenize diversity in a variety of systems (Devictor *et al*., 2008; Cao and Natuhara, 2020), and historical datasets on parasite communities should be paired with datasets on natural and anthropogenic change (e.g. climate, pollution and host abundance and fishing pressures) to identify the major driver of the observed changes in this host–parasite system. Investigating other host–parasite systems to test whether there are similar changes in beta diversity at these points in time is also recommended.

Strong correlations between species richness and host size were found. Larger fish had significantly more parasite species (Appendix Table A2), lending support to the theory that parasite diversity positively scales with host body size (Poulin, 1997). Larger hosts may support higher parasite diversity because they provide more habitat and greater niche space for parasites, they consume more prey carrying intermediate parasite life stages, and, as larger hosts are often older hosts, they have had more time to accumulate more parasites than smaller (and younger) hosts (Poulin, 1997). While conflicting relationships between host body size and parasite diversity have been found in earlier studies (Poulin, 1995), Poulin (1997) notes that the positive correlation between parasite diversity and host body size is particularly strong in fish hosts. Even though English Sole were haphazardly chosen around the average size in the UWFC, there was a significant increase in host body size with time (estimate ± standard error = 0.00118 ± 0.000350, *t* value = 3.367, *P* value = 0.00105). This relationship was statistically controlled for.

While natural history collections remain an invaluable source of historical data, the specimens they curate may not be a representative sample of their populations of origin (Tarli *et al*., 2018; Harmon *et al*., 2019). Specimens may have been collected under varying collection protocols and with collector bias, including in areas with easy researcher access (Tarli *et al*., 2018). Fortunately, many of the specimens in UWFC were collected during research cruises and preserved *via* fixation in buffered formalin and then transferred and stored in 70% ethanol. Previous work has demonstrated that these preservation techniques do not affect parasite detectability (Fiorenza *et al*., 2020), so preservation was unlikely to have impacted the results. Finally, the possibility of collectors preferentially retaining certain sizes of hosts was corrected for by using similarly sized fish across the study period.

No significant changes in the parasite richness or community dispersion of 109 English Sole were detected over the past 90 years, though there were differences in community composition over time. Multiple changes in community structure were found over the past nine decades, both between and within decades. In particular, shifts in dissimilarity due to balanced variation and unidirectional gradients were detected around the mid-1900s. While potential drivers were not explored here, anthropogenic pressures like fishing and pollution are likely to partially explain these results. Additional investigations into the historical parasite communities of the Salish Sea are encouraged, particularly those that identify the natural and anthropogenic factors that may have modified these parasite communities and those that include historical data on additional host–parasite systems.

## Data Availability

The dataset and R code are available at https://github.com/wood-lab/Preisser-et-al.-Parasitology2022.

## Supplementary material

For supplementary material accompanying this paper visit https://doi.org/10.1017/S0031182022000233. click here to view supplementary material

## Appendix A1

**Table A1.** Parasite species collected from 109 English Sole in the Puget Sound in Washington between 1930 and 2019

Species	Prevalence (%)	Mean intensity (range)
Nematodes		
*Capillaria parophrysi*	8.26	4.5 (1–14)
*Clavinema mariae*	13.76	4.6 (1–26)
*Cucullanus annulatus*	82.57	15.99 (1–136)
*Contracaecum* sp. 1	30.28	2.27 (1–10)
*Contracaecum* sp. 2	0.92	1 (1)
Spirurida sp. 1	9.17	4.8 (1–17)
Spirurida sp. 2	0.92	1 (1)
Oxyurida sp. 1	0.92	1 (1)
Nematoda spp. (unknown)	20.18	
Nematoda spp. (encysted)	8.26	
Cestodes		
Trypanorhyncha sp.	1.83	16.5 (1–32)
Trematodes		
Metacercaria sp. 1	23.85	5.04 (1–72)
Metacercaria sp. 2	25.69	Presence/absence
*Derogenes varicus*	0.92	2 (2)
*Tubulovesicula lindbergi*	3.67	3.75 (1–8)
*Lepidapedon calli*	2.75	3.67 (1–8)
Opecoelidae sp. 1	9.17	5 (1–17)
Trematoda sp. 2	4.59	3.4 (1–5)
Trematoda sp. 3	6.42	12.71 (1–39)
Trematoda spp. (unknown)	2.75	
Trematoda spp. (immature)	1.84	
Acanthocephalans		
*Echinorhynchus* spp.	18.35	2 (1–6)
Copepods		
*Acanthochondria dojirii*	1.83	1 (1)
Copepoda sp. 1	0.92	1 (1)
Copepoda sp. 2	0.92	1 (1)
Copepoda sp. 3 (chalimis)	5.50	2 (1–4)
Leeches		
*Oceanobdella pallida*	9.17	1.8 (1–5)

We report the prevalence (defined as the ratio of infected hosts to the total number of hosts examined, presented as a percentage), mean intensity (the mean number of parasites in infected hosts) and intensity range (the range of the number of parasites in each infected host) for each parasite species; all terms follow Bush *et al*. (1997). Some specimens could not be identified, either because they were nondescript juvenile stages or because specimens were not vouchered for identification (59 larval or unvouchered nematodes, 285 larval or unvouchered encysted nematodes and 9 immature or unvouchered trematodes); we report their prevalence here.

**Table A2.** Results of the generalized linear mixed-effects model (using penalized quasi-likelihood and including a temporal autocorrelation structure term) examining the relationships between species richness and time and host body size

Variable	Coefficient ± s.e.	Degrees of freedom	*t* Value	*P* value	Conditional *R*^2^	Marginal *R*^2^
**Intercept**	3.72±4.21	76	0.884	0.379	0.02605249	0.02605246
**Collection year**	−0.00161±0.00216		−0.748	0.457		
**Host body size**	0.00320±0.00151		2.12	0.0371		

Bolded rows represent significant variables in the models. Conditional and marginal *R*^2^ were calculated using the ‘rsquared’ function (package ‘piecewiseSEM’, Lefcheck, 2016). The conditional *R*^2^ is the variance explained by the entire model, while the marginal *R*^2^ is the variance explained by the fixed effects; the difference (3 × 10^−8^) is the variance explained by the random effect.

**Table A3.** Observed and estimated parasite species richness (with standard error) of the parasite community within each decade

Site	Diversity	Observed	Estimator	Standard error
1930s	Species richness	11	11.248	0.724
1940s	Species richness	10	10.995	1.862
1950s	Species richness	10	11.983	3.713
1960s	Species richness	11	15.48	7.163
1970s	Species richness	12	18.228	7.527
1980s	Species richness	10	11.988	3.72
1990s	Species richness	10	10.997	2.26
2000s	Species richness	10	10.998	1.868
2010s	Species richness	6	6.993	2.206

Parasite communities from individual fish within each decade were combined into one community per decade and a rarefaction curve was created for each decade (Fig. A6; ‘iNEXT’ function in the ‘iNEXT’ package; Chao *et al*., 2014, Hsieh *et al*., 2020).

**Table A4.** PERMANOVA and ANOVA results to determine if parasite communities of the 1930s, 1940s, 1950s, 1980s, 1990s, 2000s and 2010s decade differed in their community composition and dispersion

PERMANOVA Do decades vary in their community composition?			ANOVA Do decades vary in their dispersion of parasite communities?	
*F* statistic	*R*^2^	*P* value	*F* statistic	*P* value
**3.834**	**0.0447**	**0.002**	1.851	0.100

Parasite communities from the 1960s and 1970s were excluded as they did not have sufficient sampling of parasite diversity. Significant results are in bold.

**Table A5.** Total parasite beta diversity (dissimilarity) and its two components, balanced variation and unidirectional gradients (Baselga, 2013*a*), were calculated from the comparison of parasite communities of sequential decades using abundance data

Comparison	Balanced variation	Unidirectional gradients	Total dissimilarity
1930s–1940s	0.216	0.200	0.416
1940s–1950s	0.554	0.0956	0.649
1950s–1980s	0.537	0.0642	0.601
1980s–1990s	0.163	0.278	0.441
1990s–2000s	0.204	0.208	0.412
2000s–2010s	0.233	0.443	0.676
1930s–1940s	0.216	0.194	0.410
1940s–1950s	0.550	0.0947	0.645
1950s–1980s	0.533	0.0638	0.597
1980s–1990s	0.152	0.285	0.437
1990s–2000s	0.201	0.210	0.411
2000s–2010s	0.233	0.443	0.676

Total pairwise dissimilarity was calculated using the Bray–Curtis dissimilarity index, with estimates ranging from 0 (communities are identical) to 1 (communities are totally dissimilar). Balanced variation and unidirectional gradients also range from 0 (no balanced variation and unidirectional gradients) to the total dissimilarity value. See Fig. 1 for a plot of these values over time. These comparisons excluded unknown parasites and one species of metacercaria where only presence/absence data were available.

**Table A6.** Total parasite beta diversity (dissimilarity) and its two components, balanced variation and unidirectional gradients (Baselga, 2013*a*), were calculated among individuals within each decade to explore how community dissimilarity changed over time; these comparisons used abundance data

Decade	Balanced variation	Unidirectional gradients	Total dissimilarity
1930s	0.829	0.102	0.931
1940s	0.693	0.195	0.888
1950s	0.744	0.150	0.894
1980s	0.570	0.285	0.855
1990s	0.647	0.244	0.891
2000s	0.557	0.334	0.891
2010s	0.605	0.268	0.873
*Without singletons*			
1930s	0.832	0.0999	0.931
1940s	0.689	0.198	0.886
1950s	0.728	0.163	0.891
1980s	0.566	0.288	0.854
1990s	0.654	0.236	0.890
2000s	0.561	0.328	0.889
2010s	0.607	0.265	0.873

Total multiple site dissimilarity (Baselga, 2013*b*) was calculated using the Bray–Curtis dissimilarity index, with estimates ranging from 0 (communities are identical) to 1 (communities are totally dissimilar). Balanced variation and unidirectional gradients also range from 0 (no balanced variation and unidirectional gradients) to the total dissimilarity value. See Fig. 1 for a plot of these values over time. These comparisons excluded parasites that could not be identified to a morphotype, one species of metacercaria where only presence/absence data were available, and hosts with no parasites.

## Appendix A2

### Methods

#### Data collection

Parasites were collected with standard visual parasite detection techniques. Briefly, we examined each individual under a stereomicroscope and collected their ecto- and endoparasites. The fins and outer surface of the fish were examined with a strong light. The gills were removed from the blind side of the sole, placed in a vial with 70% ethanol, agitated to dislodge parasites and the contents of the vial were searched. We then made a ventral incision from the anus to the isthmus of the gills and removed the internal organs. To find endoparasites, we individually squashed the liver, heart, kidneys and gonads between glass plates, and searched the gastrointestinal tract and its contents. During parasite collection, we sorted parasites by morphotype, counted each morphotype and photographed a representative specimen and compiled a photographic key to aid dissectors in accurate morphotype classification. All parasites were removed from their hosts and stored in 70% ethanol.

Parasites were morphologically keyed out to the lowest possible taxonomic level by JMK (helminths) and KLL (copepods and leech) using standard parasitological keys (Yamaguti, 1971*a*, 1971*b*; Anderson *et al*., 1974; Burreson, 1977; Khalil *et al*., 1994; Gibson *et al*., 2002; Jones *et al*., 2005; Bray *et al*., 2008). Trematodes and cestodes (Phylum Platyhelminthes) were stained and mounted on glass slides according to standard techniques for long-term preservation and storage (Cable, 1977). Nematodes (Phylum Nematoda) and acanthocephalans (Phylum Acanthocephala) were cleared in lacto-phenol for identification. All parasites, excluding trematodes and cestodes, were stored in 70% ethanol for long-term preservation and storage.

After dissection, the organs and gills of each fish were placed into a vial with 70% ethanol; the vial and the fish were labelled with a matching, unique identification number and returned to the original collection jar. The parasites from each individual fish were also labelled with the species identification and the host's catalogue number and will be accessioned into the Harold W. Manter Laboratory of Parasitology at the University of Nebraska-Lincoln in Lincoln, Nebraska. The fish catalogue information at the Burke's Ichthyology Collection will be updated to include links to the parasite collections.

We could not identify some parasite specimens that were either larval and nondescript or they were not vouchered or photographed (Nematoda spp. and Trematoda spp. in Supplementary Table 1; labelled as ‘immature_trem’, ‘unknown_trem’, ‘unknown_nem’ and ‘cyst_nem’ in the Supplementary Datasheet). Conservatively, we assumed these parasites to be species already represented in these hosts and excluded them from diversity analyses, with three exceptions. Three hosts had only young, non-reproductive, unidentifiable trematodes present (labelled as ‘immature_trem’ in Supplementary Datasheet); these hosts had no other trematode species present, so these immature trematodes were included in the species richness counts for these three hosts, as there was no chance of mistakenly counting a trematode species twice. However, all unidentified parasites were excluded from beta diversity analyses. Metacercaria sp. 2 (Supplementary Table 1) was also excluded from beta diversity analyses, because only presence/absence data, not abundance, were available; these encysted metacercariae were clumped together on the fins of infected fish and could not be individually counted without destructively sampling the fin tissue.

#### Spatial and temporal autocorrelation and overdispersion

We first created a linear model with our primary predictor variable of interest (i.e. the year the fish was collected) with a Poisson distribution (equation (A1)). We tested for spatial autocorrelation using Moran's *I* on the simulated residuals of the model using the jittered coordinates of each collection locality (‘testSpatialAutocorrelation’ function in ‘DHARMa’ package; Hartig, 2018). Tests for spatial autocorrelation do not allow repeated coordinate locations as, theoretically, the same locations should have the same response variable value; however, with parasite communities, different hosts collected from the same location often have different parasite communities. We jittered the coordinates (‘jitter’ function in the ‘base’ package; R Core Team, 2020) to add a small amount of random noise to each coordinate combination to make each coordinate combination unique.

A1

We again used equation (A1) and tested for temporal autocorrelation using the Durbin–Watson test on the model residuals (‘dwtest’ function in the ‘lmtest’ package; Zeileis and Hothorn, 2002).

We tested for overdispersion of the species richness data using the function ‘dispersiontest’ in the ‘AER’ package (Kleiber and Zeileis, 2008).

#### Power analysis

We parameterized our simulations with information from our study (sample size, collection year and average richness in the earliest decade). We used the average parasite species richness in the earliest decade as the richness in the earliest year (1930) and then determined the expected average parasite species in all subsequent years under different hypothetical linear changes in richness over the 90-year period. Using these expected average parasite richness values, we then randomly simulated a potential observed parasite richness using a Poisson distribution. We used the simulated data to run generalized linear models with a Poisson error distribution, which either included collection year as a fixed factor or excluded it (null model). We compared the two models with Akaike information criterion and recorded whether the year model was better than the null model. This process was repeated 1000 times for each hypothetical change in parasite richness over time. Using these simulations, we calculated the proportion of simulations where the best model was the model that contained year as a fixed effect to determine the power of the analysis.

#### Rarefaction and extrapolation curve

We created a site-by-species matrix of individual host communities with individual hosts as the sites (rows), parasite species as the columns and the abundance of parasite species in each cell. Aggregating the data by decade and summing the abundances of each parasite species, we created a rarefaction and extrapolation curve for each decade using the ‘iNEXT’ function in the ‘iNEXT’ package (Chao *et al*., 2014; Hsieh *et al*., 2020). Results are presented in Appendix Table A3 and Fig. A6.

##### Do decades vary in their parasite assemblages?

We created a site-by-species matrix of individual host communities with individual hosts as the sites (rows), parasite species as the columns and the abundance of parasite species in each cell. We excluded fish from decades that were not sufficiently sampled. We then created a distance matrix using the Bray–Curtis dissimilarity index (‘vegdist’ function in ‘vegan’ package; Oksanen *et al*., 2019).

##### How has the composition of parasite assemblages changed across time?

For the decadal community analysis, we combined the helminth communities from individuals collected within each decade and generated a site-by-species matrix for each decade where parasite diversity was sufficiently sampled. Within each decade matrix, the entire Salish Sea served as the site (one row), and we created a column for each parasite species with the abundance of each parasite species summed across all individuals within each decade in each cell.

To analyse how the dissimilarity of individual parasite communities within each decade varied over time, we first created a site-by-species matrix for each decade, this time with individual fish representing sites (6–16 rows per matrix) and with each parasite species in a column. A few individuals had zero parasites and they were excluded from the analysis as they had no parasite community to compare.

### Results

#### Spatial and temporal autocorrelation and overdispersion

Spatial autocorrelation was not detected in the residuals (observed = 0.0732590, expected = −0.0092593, s.d. = 0.0706720, *P* = 0.243).

Temporal autocorrelation was detected in the residuals (DW = 1.62, *P* value = 0.0362). We included a temporal correlation term in the model to account for this [‘correlation = corCAR1()’ from the package ‘nlme’; Pinheiro *et al*., 2020].

The species richness data were not overdispersed (*z* = −53.245, *α* = −0.9633028, *P* = 1).
