# Supplementary material for: Parasite communities in English Sole (Parophrys vetulus) have changed in composition but not richness in the Salish Sea, Washington, USA since 1930
Source: Parasitology. 2022 Mar 3;149(6):786–98. doi: 10.1017/S0031182022000233 (PMC10090603; doi:10.1017/S0031182022000233)
Supplement: Supplementary file 1 [file S0031182022000233sup.zip › S0031182022000233sup002.docx]

library(googledrive)

library(vegan)

library(lmtest)

library(DHARMa)

library(lme4)

library(geoR)

library(spacetime)

library(gstat)

library(sf)

library(sp)

library(nlme)

library(MASS)

library(betapart)

library(dplyr)

library(ggplot2)

library(patchwork)

library(gdm)

library(vegan)

library(piecewiseSEM)

library(MuMIn)

library(caret)

library(tidyverse)

library(lmtest)

library(lme4)

library(AER)

library(asbio)

library(cowplot)

library(ape)

library(reshape2)

library(iNEXT)

library(vegan)

library(ggplot2)

library(grid)

library(viridis)

Datasheet1<-read.csv("Preisser_Dataset.csv", header=TRUE)

#Jitter coordinates to remove repeats - required for autocorrelation analyses

LatJitt<-jitter(Datasheet1$Latitude, factor=0.1, amount=NULL)

LongJitt<-jitter(Datasheet1$Longitude, factor=0.1, amount=0) #having some trouble getting same number of decimal points in longitude - restricted due to being a neg high number?

#changing from amount=NULL to amount=0 seems to fix this by changing the numbers more

#bind jittered coordinates to datasheet

Datasheet<-cbind(Datasheet1, LongJitt, LatJitt)

#convert abundance to presence/absence

datasheetPA <- decostand(x=Datasheet[,9:35], method="pa")

#add PA matrix to datasheet

Datasheet2<-cbind(Datasheet, datasheetPA)

#sum PA rows to get richness for each individual and add to datasheet - not summing unknown trems, immature trems, unknown nems, cyst nems

divtest<-rowSums(Datasheet2[,38:60])

Datasheet2$SpRich<-divtest

#UW 026118_01, UW 110086_01, UW 155836_01, UW 5523_03

#UW 2269_01

#increasing sp richness to reflect cases where unknown/immature trems represent

#the only trematode species in that host - 17077_01, 17851_02, 2269_01

Datasheet2$HostID[73]

Datasheet2$HostID[77]

Datasheet2$HostID[79]

Datasheet2$SpRich[73]<-Datasheet2$SpRich[73]+1

Datasheet2$SpRich[77]<-Datasheet2$SpRich[77]+1

Datasheet2$SpRich[79]<-Datasheet2$SpRich[79]+1

#P/A only datasheet w/ richness

DatasheetRich<-Datasheet2[,c(1:7,36:65)]

DatasheetRich$SpRich

###Polynomial equation testing

ggplot(DatasheetRich, aes(Year, SpRich) ) +

geom_point() +

stat_smooth()

lm(SpRich ~ poly(Year, 6, raw = TRUE), data = DatasheetRich) %>%

summary()

lm(SpRich~poly(Year,4)+poly(SL,4), Datasheet2) %>%

summary()

####check for temporal autocorrelation

RichLM<-glm(SpR2 ~ Year2,

family=poisson(link = "log"))

summary(RichLM)

time<-Year2

RichDW<-dwtest(RichLM, order.by = time, alternative = "two.sided", iterations = 1000,

exact =FALSE, tol = 1e-10)

RichDW

####check for spatial autocorrelation

simulationoutput3<-simulateResiduals(fittedModel=RichLM)

testSpatialAutocorrelation(simulationOutput = simulationoutput3,

x=Datasheet2$LongJitt, y=Datasheet2$LatJitt)

####################YES TEMPORAL AUTOCORRELATION in residuals (didn't test raw data)

####################NO SPATIAL AUTOCORRELATION in residuals (didn't test raw data)

##test for overdispersion

Datasheet2AB<-Datasheet2[,c(1,65)]

Datasheet2AB

rd <- glm(SpRich ~ ., data = Datasheet2AB, family = poisson)

dispersiontest(rd,trafo=1)

#data: rd

#z = -53.245, p-value = 1

#alternative hypothesis: true alpha is greater than 0

#sample estimates:

# alpha

#-0.9633028

##Accounting for temporal autocorrelation

sites1<-paste(Datasheet2$Latitude,Datasheet2$Longitude)

test.mod2<-glmmPQL(SpRich~Year+SL,random=~1+Year|sites1, data=Datasheet2,

family=poisson(link = "log"), correlation = corAR1(), verbose=FALSE)

summary(test.mod2)

##Making partial residual plot

#to model relationship between richness and year (partial regression plot) holding all other variables constant

#run original model with everything except the variable of interest

test.mod2a<-test.mod<-glmmPQL(SpRich~SL,random=~1|sites1, data=Datasheet2,

family=poisson(link = "log"), correlation=corCAR1(), verbose=FALSE)

#run new model with variable of interest against everything else

test.mod2b<-test.mod<-glmmPQL(Year~SL,random=~1|sites1, data=Datasheet2,

family=poisson(link = "log"), correlation=corCAR1(), verbose=FALSE)

#get residuals of each model

resid.2a<-residuals(test.mod2a)

resid.2b<-residuals(test.mod2b)

x <- resid.2b

y <- resid.2a

jpeg(file="PartialRegressionPlot.jpeg",

width=8, height=6, units="in", res=300)

plot(x,y,type="n",

xlab="Residuals from Model 2", ylab="Residuals from Model 1")

part <- lm(y~x)

wx = par("usr")[1:2]

new.x = seq(wx[1],wx[2],len=100)

pred = predict(part, new=data.frame(x=new.x), interval="conf")

lines(new.x,pred[,"fit"],lwd=2)

lines(new.x,pred[,"lwr"],lty=3)

lines(new.x,pred[,"upr"],lty=3)

points(x,y,pch=16,col="black")

dev.off()

#####checking for relationship between host body size and time

hist((Datasheet2$SL))

Size<-log10(Datasheet2$SL)

Size2<-Datasheet2$SL

Year2<-Datasheet2$Year

HostSLMod<-glm(Size ~ Year2)

summary(HostSLMod)

plot(Size2, Year2)

colnames(Datasheet2)

DatasheetSize<-Datasheet2[,c(5,7)]

DatasheetSize

ggplot(DatasheetSize, aes(Year, SL) ) +

geom_point() +

stat_smooth()

#########Splitting up dataset into decades for beta diversity analyses

#all decades except two (60s and 70s) reach or are close to the asymptote

#and are within 2 species of the total estimated diversity for each decade (via iNext)

Datasheet2_1930<-Datasheet2[Datasheet2$Year < 1940,] #14 #14

Datasheet2_1940<-Datasheet2[Datasheet2$Year < 1950 & Datasheet2$Year > 1939,] #14 #14

Datasheet2_1950<-Datasheet2[Datasheet2$Year < 1960 & Datasheet2$Year > 1949,] #6 #6

Datasheet2_1960<-Datasheet2[Datasheet2$Year < 1970 & Datasheet2$Year > 1959,] #8 #8

Datasheet2_1970<-Datasheet2[Datasheet2$Year < 1980 & Datasheet2$Year > 1969,] #11 #11

Datasheet2_1980<-Datasheet2[Datasheet2$Year < 1990 & Datasheet2$Year > 1979,] #13 #13

Datasheet2_1990<-Datasheet2[Datasheet2$Year < 2000 & Datasheet2$Year > 1989,] #13 #13

Datasheet2_2000<-Datasheet2[Datasheet2$Year < 2010 & Datasheet2$Year > 1999,] #16 #16

Datasheet2_2010<-Datasheet2[Datasheet2$Year > 2009,] #14 #14

Datasheet2_1930$Decade.1=1930

Datasheet2_1940$Decade.1=1940

Datasheet2_1950$Decade.1=1950

Datasheet2_1960$Decade.1=1960

Datasheet2_1970$Decade.1=1970

Datasheet2_1980$Decade.1=1980

Datasheet2_1990$Decade.1=1990

Datasheet2_2000$Decade.1=2000

Datasheet2_2010$Decade.1=2010

Datasheet3D<-rbind(Datasheet2_1930,Datasheet2_1940,Datasheet2_1950,Datasheet2_1960,

Datasheet2_1970, Datasheet2_1980, Datasheet2_1990, Datasheet2_2000,

Datasheet2_2010)

###Rarefaction

#removing all columns except break and species abundance (removed unknowns and metacercaria_sp2)

Datasheet3.2D<-Datasheet3D[,c(66,9:14,16:31)]

##aggregate based on decade

Datasheet3.2D_agg<-aggregate(. ~ Decade.1, Datasheet3.2D, sum)

rownames(Datasheet3.2D_agg)<-Datasheet3.2D_agg[,1]

Datasheet3.2D_agg[,1]<-NULL

testinext3D<-iNEXT((t(Datasheet3.2D_agg)), q = 0, datatype = "abundance", size = NULL, endpoint = NULL,

knots = 40, se = TRUE, nboot = 50)

ggiNEXT(testinext3D, type = 1, se = TRUE, facet.var = "none",

color.var = "none", grey = FALSE)

####Remove 60s and 70s from datasheet as they didn't reach their asymptote

Datasheet3E<-rbind(Datasheet2_1930,Datasheet2_1940,Datasheet2_1950,Datasheet2_1980,

Datasheet2_1990, Datasheet2_2000,Datasheet2_2010)

Datasheet3.2E<-Datasheet3E[,c(66,9:14,16:31)]

Datasheet3.2E_agg<-aggregate(. ~ Decade.1, Datasheet3.2E, sum)

rownames(Datasheet3.2E_agg)<-Datasheet3.2E_agg[,1]

Datasheet3.2E_agg[,1]<-NULL

####Beta Diversity Analyses - Between time periods

BetaDiv_Btwn<-beta.pair.abund(Datasheet3.2E_agg, index.family = "bray")

Bray_Btwn_3040<-BetaDiv_Btwn$beta.bray[1]

Bray_Btwn_4050<-BetaDiv_Btwn$beta.bray[7]

Bray_Btwn_5080<-BetaDiv_Btwn$beta.bray[12]

Bray_Btwn_8090<-BetaDiv_Btwn$beta.bray[16]

Bray_Btwn_9000<-BetaDiv_Btwn$beta.bray[19]

Bray_Btwn_0010<-BetaDiv_Btwn$beta.bray[21]

Bray_Btwn<-c(Bray_Btwn_3040,Bray_Btwn_4050,Bray_Btwn_5080,

Bray_Btwn_8090,Bray_Btwn_9000,Bray_Btwn_0010)

Uni_Btwn_3040<-BetaDiv_Btwn$beta.bray.gra[1]

Uni_Btwn_4050<-BetaDiv_Btwn$beta.bray.gra[7]

Uni_Btwn_5080<-BetaDiv_Btwn$beta.bray.gra[12]

Uni_Btwn_8090<-BetaDiv_Btwn$beta.bray.gra[16]

Uni_Btwn_9000<-BetaDiv_Btwn$beta.bray.gra[19]

Uni_Btwn_0010<-BetaDiv_Btwn$beta.bray.gra[21]

Uni_Btwn<-c(Uni_Btwn_3040,Uni_Btwn_4050,Uni_Btwn_5080,

Uni_Btwn_8090,Uni_Btwn_9000,Uni_Btwn_0010)

Bal_Btwn_3040<-BetaDiv_Btwn$beta.bray.bal[1]

Bal_Btwn_4050<-BetaDiv_Btwn$beta.bray.bal[7]

Bal_Btwn_5080<-BetaDiv_Btwn$beta.bray.bal[12]

Bal_Btwn_8090<-BetaDiv_Btwn$beta.bray.bal[16]

Bal_Btwn_9000<-BetaDiv_Btwn$beta.bray.bal[19]

Bal_Btwn_0010<-BetaDiv_Btwn$beta.bray.bal[21]

Bal_Btwn<-c(Bal_Btwn_3040,Bal_Btwn_4050,Bal_Btwn_5080,

Bal_Btwn_8090,Bal_Btwn_9000,Bal_Btwn_0010)

Decade_Comparison<-c(1:6)

BetaDiv_Btwn_Bray<-as.data.frame(cbind(Decade_Comparison,Bray_Btwn,Uni_Btwn,Bal_Btwn))

BetaDiv_Btwn_Bray_melt<-melt(BetaDiv_Btwn_Bray, id.vars="Decade_Comparison")

color=c("black", "#009E73", "#0072B2")

jpeg(file="DissimilarityAbundBtwnDecades_Apr28.jpeg",

width=8, height=6, units="in", res=300)

Between1<-ggplot(BetaDiv_Btwn_Bray_melt, aes(x=Decade_Comparison))+

geom_point(aes(y = value, color = variable), shape=15, size=3) +

geom_line(aes(y = value, color = variable)) +

labs(y="Dissimilarity", x="", col="Beta Diversity Measure")+

ggtitle("Balanced Variation, Unidirectional Gradients, and

Total Dissimilarity Between Time Periods")+

labs(x="Decade Comparison", y="Dissimilarity",

col="Beta Diversity Measure")+

theme(plot.title = element_text(hjust = 0.5),

panel.background = element_blank(),axis.text.x=element_blank(),

axis.ticks.x = element_blank(),legend.position = "none") + labs(tag = "A") +

scale_color_manual(values = c("black","#009E73", "#0072B2"))

dev.off()

#"#009E73", "#0072B2"

####Beta Diversity Analyses - Within time periods

colnames(Datasheet2_1930)

Datasheet2_1930.1<-Datasheet2_1930[,c(1,9:14,16:31)]

Datasheet2_1940.1<-Datasheet2_1940[,c(1,9:14,16:31)]

Datasheet2_1950.1<-Datasheet2_1950[,c(1,9:14,16:31)]

Datasheet2_1980.1<-Datasheet2_1980[,c(1,9:14,16:31)]

Datasheet2_1990.1<-Datasheet2_1990[,c(1,9:14,16:31)]

Datasheet2_2000.1<-Datasheet2_2000[,c(1,9:14,16:31)]

Datasheet2_2010.1<-Datasheet2_2010[,c(1,9:14,16:31)]

rownames(Datasheet2_1930.1) <- Datasheet2_1930.1[,1]

Datasheet2_1930.1[,1] <- NULL

rownames(Datasheet2_1940.1) <- Datasheet2_1940.1[,1]

Datasheet2_1940.1[,1] <- NULL

rownames(Datasheet2_1950.1) <- Datasheet2_1950.1[,1]

Datasheet2_1950.1[,1] <- NULL

rownames(Datasheet2_1980.1) <- Datasheet2_1980.1[,1]

Datasheet2_1980.1[,1] <- NULL

rownames(Datasheet2_1990.1) <- Datasheet2_1990.1[,1]

Datasheet2_1990.1[,1] <- NULL

rownames(Datasheet2_2000.1) <- Datasheet2_2000.1[,1]

Datasheet2_2000.1[,1] <- NULL

rownames(Datasheet2_2010.1) <- Datasheet2_2010.1[,1]

Datasheet2_2010.1[,1] <- NULL

Bray_TP30<-beta.multi.abund(Datasheet2_1930.1, index.family="bray")

Bray_TP40<-beta.multi.abund(Datasheet2_1940.1, index.family="bray")

Bray_TP50<-beta.multi.abund(Datasheet2_1950.1, index.family="bray")

Bray_TP80<-beta.multi.abund(Datasheet2_1980.1, index.family="bray")

Bray_TP90<-beta.multi.abund(Datasheet2_1990.1, index.family="bray")

Bray_TP00<-beta.multi.abund(Datasheet2_2000.1, index.family="bray")

Bray_TP10<-beta.multi.abund(Datasheet2_2010.1, index.family="bray")

Decade_W<-as.vector(c(1930, 1940, 1950, 1980, 1990, 2000, 2010))

Bray_Within30<-Bray_TP30$beta.BRAY

Bray_Within40<-Bray_TP40$beta.BRAY

Bray_Within50<-Bray_TP50$beta.BRAY

Bray_Within80<-Bray_TP80$beta.BRAY

Bray_Within90<-Bray_TP90$beta.BRAY

Bray_Within00<-Bray_TP00$beta.BRAY

Bray_Within10<-Bray_TP10$beta.BRAY

Uni_Within30<-Bray_TP30$beta.BRAY.GRA

Uni_Within40<-Bray_TP40$beta.BRAY.GRA

Uni_Within50<-Bray_TP50$beta.BRAY.GRA

Uni_Within80<-Bray_TP80$beta.BRAY.GRA

Uni_Within90<-Bray_TP90$beta.BRAY.GRA

Uni_Within00<-Bray_TP00$beta.BRAY.GRA

Uni_Within10<-Bray_TP10$beta.BRAY.GRA

Bal_Within30<-Bray_TP30$beta.BRAY.BAL

Bal_Within40<-Bray_TP40$beta.BRAY.BAL

Bal_Within50<-Bray_TP50$beta.BRAY.BAL

Bal_Within80<-Bray_TP80$beta.BRAY.BAL

Bal_Within90<-Bray_TP90$beta.BRAY.BAL

Bal_Within00<-Bray_TP00$beta.BRAY.BAL

Bal_Within10<-Bray_TP10$beta.BRAY.BAL

Total_Bray<-as.vector(c(Bray_Within30,Bray_Within40,Bray_Within50,Bray_Within80,

Bray_Within90,Bray_Within00,Bray_Within10))

Unidirectional_Gradients<-as.vector(c(Uni_Within30,Uni_Within40,Uni_Within50,Uni_Within80,

Uni_Within90,Uni_Within00,Uni_Within10))

Balanced_Variation<-as.vector(c(Bal_Within30,Bal_Within40,Bal_Within50,Bal_Within80,

Bal_Within90,Bal_Within00,Bal_Within10))

BetaDiv_Within_Bray<-as.data.frame(cbind(Decade_W, Total_Bray, Unidirectional_Gradients, Balanced_Variation))

BetaDiv_Within_Bray_Melt<-melt(BetaDiv_Within_Bray, id.vars="Decade_W")

jpeg(file="DissimilarityAbundWithinDecades_Apr28.jpeg",

width=8, height=6, units="in", res=300)

Within1<-ggplot(BetaDiv_Within_Bray_Melt, aes(x=Decade_W))+

geom_point(aes(y = value, color = variable), shape=15, size=3) +

geom_line(aes(y = value, color = variable)) +

labs(y="Dissimilarity", x="", col="Beta Diversity Measure")+

ggtitle("Balanced Variation, Unidirectional Gradients, and

Total Dissimilarity Between Time Periods")+

labs(x="Decade", y="Dissimilarity",

col="Beta Diversity Measure")+

theme(plot.title = element_text(hjust = 0.5),

panel.background = element_blank(),axis.text.x=element_blank(),

axis.ticks.x = element_blank(),

legend.position = "none")+

labs(tag = "B") +

scale_color_manual(values = c("black","#009E73", "#0072B2"))

dev.off()

# extract the legend from one of the plots

legend <- get_legend(

# create some space to the left of the legend

Within + theme(legend.box.margin = margin(0, 0, 0, 2)) #get within legend by removing "legend=none" argument

)

plots<-plot_grid(Between1/Within1)

# add the legend to the row we made earlier. Give it one-third of

# the width of one plot (via rel_widths).

plot_grid(plots, legend, rel_widths = c(3,.8))

jpeg(file="DissimilarityAbund_BW_Decades_Apr28.jpeg",

width=9, height=6, units="in", res=300)

plot_grid(plots, legend, rel_widths = c(3,.8))

dev.off()

###########Make proportion bar plot

meltDatasheet3.2D<-melt(Datasheet3.2D, id.vars="Decade.1")

jpeg(file="ProportionalAbund_Apr28.jpeg",

width=8, height=6, units="in", res=300)

ggplot(meltDatasheet3.2D, aes(fill=variable, y=value, x=Decade.1))+geom_bar(position="fill", stat="identity")+

scale_fill_manual(values=c("#000000","#050E69",

"#1525CA","#5E6BF4","#959DF9","#BEC3FB","#E0E2FB","#C2F3F7","#93EDF4",

"#5AC9D2","#31B8C2","#11949E","#08646B","#4E4F0C","#84861D","#B7BA1A",

"#D7DA10","#F6FA0E","#F7F992","#EAEAC4","#CACAC8","#90908F"))+

labs(x="Decade", y="Proportional Abundance",

col="Parasite Species")+

ggtitle("Proportion of Parasite Species Abundances Across Nine Decades")+

theme(plot.title = element_text(hjust = 0), panel.background = element_blank(), axis.text.x = element_blank(),

axis.ticks.x = element_blank())

dev.off()

############Betadisper & Adonis

#remove hosts with Metacercaria sp2 (P/A only, and this analysis uses abundances) - UW 151702_08

#remove hosts where the only parasite is unknown trem (can't analyze) - UW 2269_01

#Remove fish with no parasites (UW 026118_01, UW 110086_01, UW 155836_01, UW 5523_03)

Datasheet3E_Zero<-Datasheet3E[-c(5,75,35,42,86,27),]

rownames(Datasheet3E_Zero)<-Datasheet3E_Zero[,1]

Datasheet3E_Zero[,1]<-NULL

Datasheet3E_meta<-Datasheet3E_Zero[,c(64,65)]

Datasheet3E_multi<-Datasheet3E_Zero[,c(8:13,15:30)]

######Betadisper

Bray_3E<-vegdist(Datasheet3E_multi, method="bray", binary=FALSE)

Bray_3E.bd <- betadisper(d=Bray_3E,

group=Datasheet3E_meta$Decade.1,

type="centroid", bias.adjust=TRUE)

Bray_3E.bd.anova<-anova(Bray_3E.bd)

Bray_3E.bd.anova

########Adonis

set.seed(2020)

ado.3E<-adonis(Bray_3E ~ Decade.1, data=Datasheet3E_meta)

ado.3E

sitecols3E<-c("midnightblue","blue","darkcyan","springgreen4","springgreen1",

"greenyellow","yellow")

jpeg(filename="PCoA_3E_Apr28",

width = 6, height = 4, units="in",res = 300)

plot(Bray_3E.bd, hull=F, label=F,

main=expression(""), col=sitecols3E, #was sitecols

xlab="", ylab="", pch=c(20,20,20,20,20,20,20),cex=2, sub="",

xaxt='n',yaxt='n',cex.main=2)

ordihull(Bray_3E.bd, Datasheet3E_meta$Decade.1,

draw = c("polygon"), col = sitecols3E, alpha=0.2, lwd=0.05) #was sitecols

legend("topright",title="Decade",legend=c("1930s","1940s", "1950s", "1980s",

"1990s", "2000s", "2010s"),

col=sitecols3E,pch=c(20,20,20,20,20,20,20),bg="white",ncol = 1, cex = 1, pt.cex=1.5)

mtext("betadisper = not significant", side=1, line=0.25)

mtext("adonis = significant", side=1, line=1.25)

dev.off()

###################

nrow(Datasheet2)

colnames(Datasheet2)

#Remove fish with no parasites (UW 026118_01, UW 110086_01, UW 155836_01, UW 5523_03)

#Remove fish with only unknown parasites or metacercaria sp2 (UW 2269_01, UW 151702_08)

Datasheet2.D<-Datasheet2[-c(9,30,63,103,79,56),]

Comm<-Datasheet2.D[,c(1,9:14,16:31)]

rownames(Comm)<-Comm[,1]

Comm[,1]<-NULL

Env<-Datasheet2.D[,c(1,3:5)]

rownames(Env)<-Env[,1]

Env[,1]<-NULL

Env_30<-Env[Env$Year < 1940,] #14 #14

Env_40<-Env[Env$Year < 1950 & Env$Year > 1939,] #14

Env_50<-Env[Env$Year < 1960 & Env$Year > 1949,] #6

Env_60<-Env[Env$Year < 1970 & Env$Year > 1959,] #8

Env_70<-Env[Env$Year < 1980 & Env$Year > 1969,] #11

Env_80<-Env[Env$Year < 1990 & Env$Year > 1979,] #13

Env_90<-Env[Env$Year < 2000 & Env$Year > 1989,] #13

Env_00<-Env[Env$Year < 2010 & Env$Year > 1999,] #16

Env_10<-Env[Env$Year > 2009,] #14

Env_30$Decade<-1930

Env_40$Decade<-1940

Env_50$Decade<-1950

Env_60$Decade<-1960

Env_70$Decade<-1970

Env_80$Decade<-1980

Env_90$Decade<-1990

Env_00$Decade<-2000

Env_10$Decade<-2010

Env2<-as.data.frame(rbind(Env_30,Env_40,Env_50,Env_60,Env_70,Env_80,

Env_90,Env_00,Env_10))

Bray_Cent<-vegdist(Comm, method="bray", binary=FALSE)

Bray_Cent.bd <- betadisper(d=Bray_Cent,

group=Env2$Decade,

type="centroid")

color<-c("midnightblue","blue","darkcyan","springgreen4","springgreen1","chartreuse3",

"greenyellow","darkolivegreen1","yellow")

jpeg(filename="PCoA_Centroids_6.4.jpg",

width = 6, height = 4, units="in",res = 300)

plot(Bray_Cent.bd, hull=FALSE, segments=FALSE, label=F, ellipse=TRUE, conf=0.5, pch=26, col=color)

dev.off()
